# Comparing the Effects of Oral Contraceptives Containing Levonorgestrel With Products Containing Antiandrogenic Progestins on Clinical, Hormonal, and Metabolic Parameters and Quality of Life in Women With Polycystic Ovary Syndrome: Crossover Randomized Controlled Trial Protocol

**DOI:** 10.2196/resprot.8631

**Published:** 2017-09-29

**Authors:** Mina Amiri, Fatemeh Nahidi, Davood Khalili, Razieh Bidhendi-Yarandi, Fahimeh Ramezani Tehrani

**Affiliations:** ^1^ Department of Midwifery and Reproductive Health Faculty of Nursing and Midwifery Shahid Beheshti University of Medical Sciences Tehran Islamic Republic Of Iran; ^2^ Reproductive Endocrinology Research Center Research Institute for Endocrine Sciences Shahid Beheshti University of Medical Sciences Tehran Islamic Republic Of Iran; ^3^ Prevention of Metabolic Disorders Research Center Research Institute for Endocrine Science Shahid Beheshti University of Medical Sciences Tehran Islamic Republic Of Iran; ^4^ Department of Epidemiology and Biostatistics School of Public Health Tehran University of Medical Sciences Tehran Islamic Republic Of Iran

**Keywords:** polycystic ovary syndrome, oral contraceptives, randomized controlled trial, progestin

## Abstract

**Background:**

Oral contraceptives (OCs) have been used as a first-line option for medical treatment in women with polycystic ovary syndrome (PCOS). Despite theoretical superiority of products containing antiandrogenic progestins compared to OCs containing levonorgestrel (LNG), the clinical advantage of these compounds remains unclear.

**Objective:**

The aim of this study was to compare the effects of OCs containing LNG with products containing antiandrogenic progestins including cyproterone acetate, drospirenone, and desogestrel on clinical, hormonal, and metabolic parameters and quality of life in women with PCOS.

**Methods:**

We conducted a 6-arm crossover randomized controlled trial with each arm including OCs containing LNG and one of those 3 OCs containing antiandrogenic progestins. The anthropometric and clinical manifestations and hormonal and biochemical parameters of participants were assessed at 6 time points including baseline, after washout period, and 3 and 6 months after intervention.

**Results:**

The study is ongoing and follow-up of recruited women will continue until 2018.

**Conclusions:**

This study will provide scientific evidence on comparability of OCs with the various progesterones that will assist in decision making taking into account cost effectiveness.

**Trial Registration:**

Iranian Registry of Clinical Trials IRCT201702071281N2; http://www.irct.ir/searchresult.php? keyword=&id=1281&number=2&prt=12869&total=10&m=1 (Archived by WebCite at http://www.webcitation.org/6tSP8FNWo)

## Introduction

Polycystic ovary syndrome (PCOS), the most common endocrine and metabolic disorder [1-3], which affects between 5% and 10% of reproductive age women [4,5], is characterized by chronic oligo-ovulation or anovulation and hyperandrogenism resulting in infertility, menstrual irregularities, hirsutism, acne, and alopecia [6,7]. PCOS is associated with an increased risk of metabolic disorders such as obesity, dyslipidemia, hyperinsulinemia, insulin resistance, and metabolic disturbances, which increase the risk of diabetes mellitus and cardiovascular disease [8-12]. Previous studies report that certain aspects of PCOS have negative effects on the health-related quality of life for these women [13,14].

Oral contraceptives (OCs) are frequently recommended as first-line medical treatment for the long-term management of menstrual disturbances and hyperandrogenism manifestations in women with PCOS who do not seek pregnancy [15-18]. The remedial effect of OCs is attributed to the suppression of pituitary gonadotropin secretion and a decrease in androgen secretion [19,20]; in addition, the estrogen component of these compounds increases circulating levels of sex hormone-binding globulin (SHBG), which decreases androgen bioavailability [1,21]. Moreover, the progestin component of some of the new generation of OCs inhibits 5α-reductase activity and acts as an antagonist at the androgen receptor level [21,22], theoretically resulting in an increase in antiandrogenic activity [23]. Despite theoretical advantages of OCs with antiandrogenic properties from compounds such as cyproterone acetate (CA), drospirenone (DRSP), desogestrel (DSG), and ethinyl estradiol (EE) compared to OCs containing levonorgestrel (LNG) [1,24], clinical advantages of these compounds remain unclear [25].

In addition to the antiandrogenic effect of OCs, their metabolic effect is one of the main issues in OC therapy in patients with PCOS [26]. In fact, some studies have raised concerns regarding the potential adverse cardiovascular and metabolic effects of OCs in women with PCOS [2,27-30], including worsening insulin resistance and glucose tolerance and potential adverse effects on lipid patterns. It is also unclear whether the metabolic effects of OCs on PCOS are reduced by the use of certain progestin compounds. Despite the theoretical antiandrogenic advantage of the new generation of OCs, their possible metabolic adverse effects may be a serious threat [31].

Several studies that assessed the effectiveness of OCs on clinical, biochemical, and metabolic profiles in PCOS patients have reported conflicting findings [1,10,15,16,32-35]. However, a limited number of studies have compared the effects of the various OC products on different aspects of PCOS; the majority of these studies did not assess effects of OCs containing LNG.

Accordingly, in this crossover randomized controlled clinical trial, we aimed to compare the effects of OCs containing LNG with products containing antiandrogenic progestins on the clinical, hormonal, and metabolic aspects and quality of life of reproductive-age women with PCOS.

We hypothesize that in PCOS patients, OCs containing antiandrogenic progestins including DRSP, CA, and DSG have no advantage over products containing LNG on the clinical, hormonal, and metabolic profiles of these women or on their quality of life.

## Methods

### Overview of Study Design and Procedures

**Figure 1:**
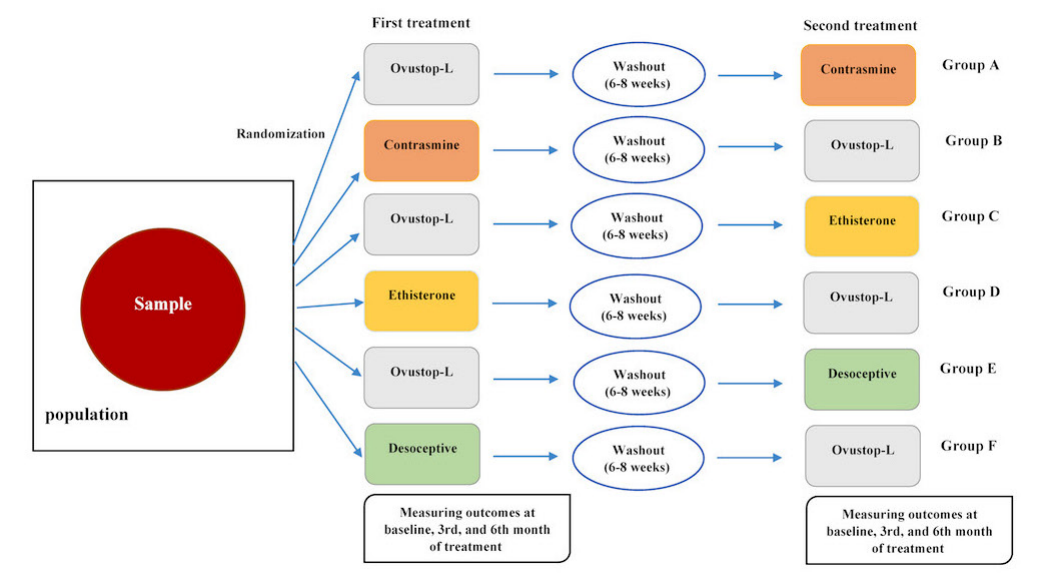
Overview of study design.

This study is a single institution crossover randomized controlled clinical trial with 6 treatment groups (A, B, C, D, E, and F) that commenced in February 2016. Our study design was based on the Consolidated Standards of Reporting Trials (CONSORT) requirements [36]. The research team included a gynecologist, midwife, endocrinologist, epidemiologist, and statistician. The trial was conducted at the endocrinology clinic of the Research Institute for Endocrine Sciences (RIES) of the Shahid Beheshti University of Medical Sciences, Tehran, Iran. Figure 1 presents an overview of the study protocol. Participants were recruited through 4 primary sources: online through professional social networks and referrals from municipal health centers, private clinics, and the Tehran Lipid and Glucose Study (TLGS). The trial was conducted at an endocrine outpatient clinic.

**Figure 2:**
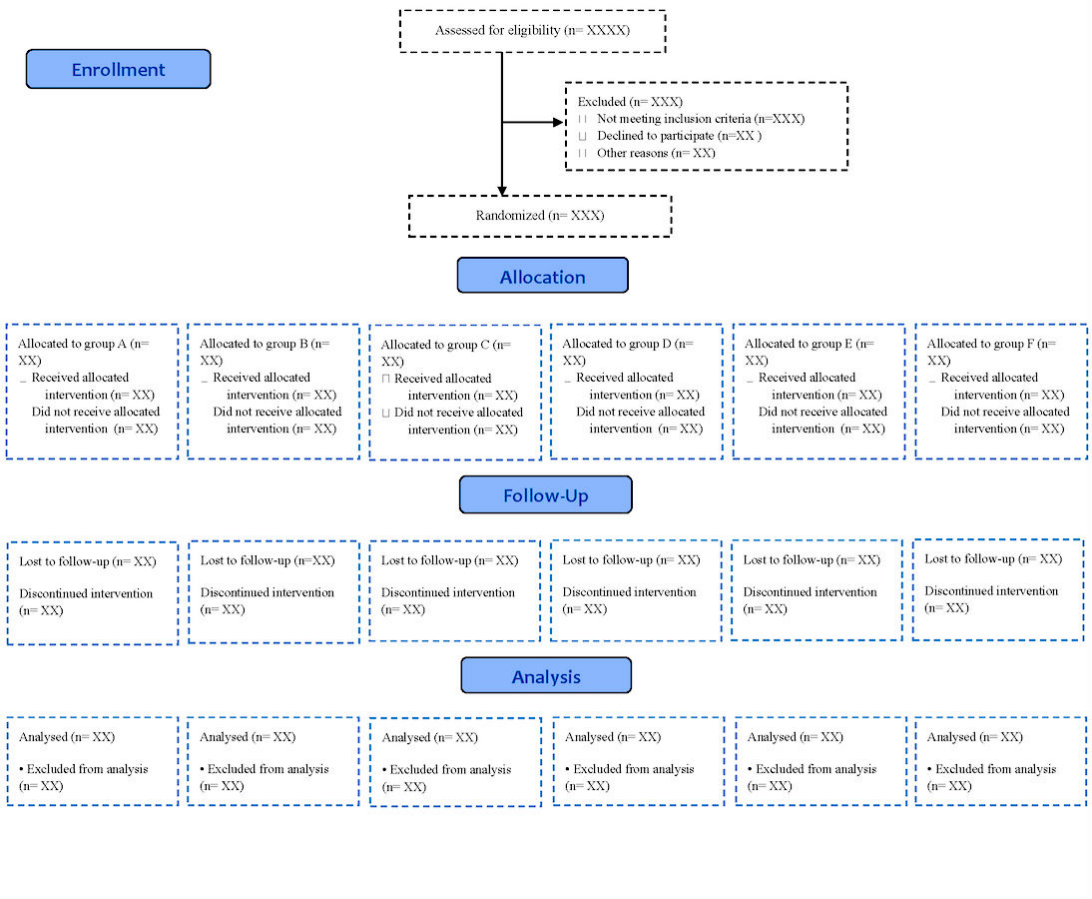
Consolidated Standard of Reporting Trials flow diagram of the study.

Before entering the study, the purpose of the protocol was clearly explained to the patients and written informed consent was obtained from all women enrolled. All eligible patients were randomly assigned to treatment groups (Figure 2). Patients in each of the groups alternately received OCs containing LNG or a product containing antiandrogenic progestins including CA, DRSP, DSG, or EE for 6 months. There was a washout period of 6 to 8 weeks between the 2 treatments. Clinical, hormonal, metabolic, and quality of life assessments were assessed at the time of recruitment and again at follow-ups. Hence, each patient was assessed for clinical and biochemical measurements at 6 time points: before the first treatment (baseline 1), after taking the first treatment for 3 months, after taking the first treatment for 6 months, 6 to 8 weeks after stopping the first treatment (baseline 2), after taking the second treatment for 3 months, and after taking the second treatment for 6 months (see Table 1).

Baseline fasting (for at least 9 hours) blood samples were collected between days 3 and 5 of the spontaneous menstrual cycle or progesterone-induced menstrual bleeding. Follow-up visits for blood sampling and clinical assessment were performed at 3 to 7 days after using last tablet. All sera were stored at –80°C until the time of testing. Following completion of the study (samples and data collection), all data will be analyzed using Stata software (StataCorp LLC).

### Ethical Considerations

Approval was obtained from the ethics committee of Shahid Beheshti University of Medical Sciences, Tehran, Iran (ethics code IR.SBMU.PHNM.1395.649). The trial is registered at the Iran Registry of Clinical Trials [IRCT201702071281N2].

Written informed consent was obtained from eligible participants after the content was clearly explained to the subjects by the trial assistant. All research tools, including questionnaires, will be completely anonymous; a unique code will, however, be included in each form in order to manage further references. All information on participants will be securely stored and only accessible to authenticated trial team members.

### Participants

Women with PCOS (age range 18 to 45 years) were recruited at the endocrine outpatient clinic of the RIES of the Shahid Beheshti University of Medical Sciences, Tehran, Iran, by publicly advertising in the community or by referral from physicians or other clinicians. PCOS was diagnosed according to the 2006 criteria from the Androgen Excess Society (AES) (hirsutism or hyperandrogenemia and oligo-ovulation/ anovulation or polycystic ovaries with exclusion of other androgen excess or related disorders) [37]. Patients testing positive for secondary etiologies including hyperprolactinemia, thyroid dysfunction, Cushing syndrome, congenital adrenal hyperplasia, and virilizing tumors were excluded from the study. Inclusion criteria were normal fasting plasma glucose (<100 mg/dL), normal cardiometabolic system, and normal hepatic function; all patients were nonsmokers and had negative pregnancy tests before enrollment. None of the women had taken medications known to affect plasma sex steroids for at least 3 months before the study. None of the patients planned to become pregnant or had had contraindications with the use of OCs.

Exclusion criteria were history of use of anexogenous hormonal agent within past 3 months; systemic disease such as cardiovascular disorder, renal disease, or liver disease; endocrinopathies including diabetes mellitus, thyroid dysfunction, hyperprolactinemia, and Cushing syndrome; contraindications to OCs; use of any medication related to PCOS such as hormonal, insulin sensitizer, or antiandrogen drugs within the previous 3 months; willingness for pregnancy; smoking; or any serious side effects of contraceptive use such as thrombosis, jaundice, or hepatic disorders. We excluded current study participants if they were unable to actively continue the cooperation required due to sickness, pregnancy, etc. Patients could terminate participation in the study for any reason. Patients who discontinued intervention for ≥2 months were excluded from the analyses.

### Interventions

In this study, patients were randomly assigned to treatment groups with interventions using different OC products:

- Group A: first treatment—EE 30 µg + LNG 0.15 mg daily for 6 months; second treatment—EE 30 µg + DRSP 3 mg daily for 6 months
- Group B: first treatment—EE 30 µg + DRSP 3 mg daily for 6 months; second treatment—EE 30 µg + LNG 0.15 mg daily for 6 months
- Group C: first treatment—EE 30 µg + LNG 0.15 mg daily for 6 months; second treatment—EE 35 µg + CA 2 mg daily for 6 months
- Group D: first treatment—EE 35 µg + CA 2 mg daily for 6 months; second treatment—EE 30 µg + LNG 0.15 mg daily for 6 months
- Group E: first treatment—EE 30 µg + LNG 0.15 mg daily for 6 months; second treatment—EE 30 µg + DSG 150 µg daily for 6 months
- Group F: first treatment—EE 30 µg + DSG 150 µg daily for 6 months; second treatment—EE 30 µg + LNG 0.15 mg daily for 6 months

All participants received EE 30 µg + LNG 0.15 mg as the standard treatment. To eliminate carryover effect of treatments, a washout period of 6 to 8 weeks was in place between 2 treatments (Figure 2). Interventions were performed by a trained midwife with the assistance of another person who was aware of the type of intervention.

### Study Outcomes

Outcome measures were collected at 6 time points: before treatments, after washout period, and after 3 and 6 months of treatments (Table 1).

Only one person conducted clinical assessments of participants, and she was blinded to groups to minimize any assessor effects. Biochemical measurements were performed by an expert laboratory technician under the supervision of a laboratory sciences specialist.

In this trial, the primary outcomes were free androgen index (FAI) and homeostasis model assessment–insulin resistance (HOMA-IR). Secondary outcomes were modified Ferriman-Gallwey score (FG); acne; regularity of menstrual cycles; blood pressure; androgenic profiles including follicle-stimulating hormone (FSH), luteinizing hormone (LH), total testosterone (tT), SHBG, androstendione (A4), and dehydroepiandrosterone sulfate (DHEAS); metabolic profiles including fasting blood sugar (FBS) fasting insulin, HOMA-IR, triglycerides (TG), total cholesterol (TC), high-density lipoprotein cholesterol (HDL-C), low-density lipoprotein cholesterol (LDL-C); and quality of life assessed by Health-Related Quality of Life Questionnaire for Polycystic Ovary Syndrome (PCOSQ-50) [13].

**Table 1:** Schedule of clinical and biochemical assessments.

Item		Baseline 1	At 3rd month of treatment	At 6th month of treatments	Baseline 2 (after washout period)	At 3rd month of treatment	At 6th month of treatment
**Demographic characteristics**		x					
	Age, marital, educational, and occupational status						
**Menstrual history**							
	Age of menarche	x					
	Last menstrual period	x	x	x	x	x	x
	Interval between menstrual cycles	x	x	x	x	x	x
	Amenorrhea history	x					
**Medical and family history**							
	Chief complaints (infertility, hirsutism, menstrual irregularity, acne, alopecia)	x					
	Duration of PCOS^a^ diagnosis	x					
	History of previous treatments	x					
	Hair removal methods	x	x	x	x	x	x
	Past medical history	x					
	Family history	x					
**Anthropometric and blood pressure measurements**							
	Height	x					
	Weight	x	x	x	x	x	x
	Waist circumference	x	x	x	x	x	x
	Hip circumference	x	x	x	x	x	x
	Wrist circumference	x	x	x	x	x	x
	Blood pressure	x	x	x	x	x	x
**Clinical hyperandrogenism symptom assessments**							
	FG^b^ score	x	x	x	x	x	x
	Acne	x	x	x	x	x	x
	Androgenic alopecia	x	x	x	x	x	x
**Laboratory assessments**							
	Hormonal parameters (FSH^c^, LH^d^, tT^e^, FAI^f^, SHBG^g^, A4^h^, DHEAS^i^)	x	x	x	x	x	x
	Metabolic parameters (FBS^j^, fasting insulin, HOMA-IR^k^, TG^l^, TC^m^, LDL-C^n^, HDL-C^o^)	x	x	x	x	x	x
Quality of life		x	x	x	x	x	x
Ultrasound assessment		x					

^a^PCOS: polycystic ovary syndrome.

^b^FG: Ferriman-Gallwey score.

^c^FSH: follicle-stimulating hormone.

^d^LH: luteinizing hormone.

^e^tT: total testosterone.

^f^FAI: free androgen index.

^g^SHBG: sex hormone-binding globulin.

^h^A4: androstenedione.

^i^DHEAS: dehydroepiandrosterone sulfate.

^j^FBS: fasting blood sugar.

^k^HOMA-IR: homeostatic model assessment–insulin resistance.

^l^TG: triglycerides.

^m^TC: total cholesterol.

^n^LDL-C: low-density lipoprotein cholesterol.

^o^HDL-C: high-density lipoprotein cholesterol.

### Clinical Assessment

In this study, a single trained midwife in the endocrinology clinic under supervision of a reproductive endocrinologist assessed the anthropometric parameters, menstrual cycles, hirsutism, acne, alopecia, and acanthosis nigricans in participants at baselines and follow-ups. She was blinded to type of treatment.

Patient weights were measured when they were minimally clothed using a digital scale (Seca 707, Seca GmbH) and rounded to the nearest 100 grams. Height was measured without shoes in the standing position with shoulders in normal alignment using a tape measure. Waist circumference was measured with an unstretched tape measure at the level of umbilicus without any pressure to the body surface and recorded to the nearest 0.1 cm. Hip circumference was measured at the level of anterior superior iliac spine without any pressure to the body surface. Wrist circumstance was measured similarly. Body mass index was calculated as weight in kilograms (kg) divided by height squared (m^2^). Systolic and diastolic blood pressure were measured twice on the right arm with the patient in a seated position by a qualified midwife with a standard mercury sphygmomanometer after the subject sat for 15 minutes; the mean of these 2 measurements was recorded.

All patients were evaluated for regularity of menstrual cycles. Those who had intervals of menstrual cycle more than 35 days were diagnosed as having oligomenorrhea, less than 22 days as polymenorrhea, and those with absence of menstrual periods for 6 months or longer as amenorrhea [37,38]. Spotting was assessed and registered.

In this study, the FG score was used for determining the density of terminal hair at 9 different body sites: upper lip, chin, chest, upper back, lower back, upper abdomen, lower abdomen, arm, and thigh; this scoring system grades excess terminal body or facial hair growth based on a scale of 0 (absence of terminal hairs) to 4 (frank virilization), and a total score of 8 or more is considered as hirsutism [39,40]. Participants were asked to refrain from shaving or using other depilatory methods in the month before evaluation to improve accuracy of assessment.

The severity of acne was classified according to a grading system based on the number of lesions and their spread on the face, back, and chest: mild—comedones (clogged hair follicles often similar to pustules that do not have any inflammation) present; moderate—the number of erythematous and popular moderate lesions was from 10 to 40 or number of pustules was from 10 to 40 (especially on the face); moderate to severe—the number of multiple and numerous papules and pustules was from 40 to 100 often accompanied by comedones in numbers of 40 to 100 on the face, upper chest, and back; severe—nodulocystic acne with painful nodules or pustules frequently observed on the chest and face and accompanied by smaller papules, pustules, and comedones [41].

To diagnose androgenic alopecia in participants, we used the following classification, which was presented by Ludwig [42] in Germany:

- Grade I: perceptible thinning of the hair on the crown limited in the front by a line situated 1 to 3 cm behind the frontal hair line
- Grade II: pronounced rarefaction of the hair on the crown within the area seen in Grade I
- Grade III: complete baldness (total denudation) within the area seen in Grades I and II

In addition, we will assess the common side effects of OCs during and after follow-ups (Table 2).

### Hormonal Assay

FSH and LH will be measured by immunoradimetric assay (IRMA) (Institute of Isotopes Co Ltd) using the Wallac Wizard gamma counter (GMI Inc); tT, A4, and DHEAS will be measured by enzyme immunoassay (EIA) (Diagnostics Biochem Canada Inc). SHBG will be measured by immunoenzymometric assay (IEMA) (Mercodia AB); all enzyme-linked immunosorbent assay (ELISA) tests were performed using the Sunrise ELISA reader (Tecan Trading AG); and FAI will be calculated using the formula [tT(nmol/L)×100/SHBG(nmol/L)].

Biochemical hyperandrogenemia will be identified if FAI, DHEAS, or A4 levels are in the upper 95th percentile (tT=0.89 ng/mL, A4=2.9 ng/mL, DHEAS=179 μg/dL, FAI=5.39) considering the women studied were not on any hormonal medication and had no clinical evidence of hyperandrogenism and menstrual dysfunction. Hyperandrogenism was determined as clinical hyperandrogenism and/or biochemical hyperandrogenemia [43].

Inter- and intra-assay coefficients of variation for all hormonal measurements will be defined.

**Table 2:** A comparison of the common side effects of oral contraceptives in polycystic ovary syndrome patients.

Side effects	Type of treatment			
	OC^a^ containing LNG^b^	OC containing CA^c^	OC containing DRSP^d^	OC containing DSG^e^
Headache	x	x	x	x
Dizziness	x	x	x	x
Nausea	x	x	x	x
Vomiting	x	x	x	x
Breast pain/tenderness	x	x	x	x
Spotting	x	x	x	x

^a^OC: oral contraceptive.

^b^LNG: levonorgestrel.

^c^CA: cyproterone.

^d^DRSP: drospirenone.

^e^DSG: desogestrel.

### Metabolic Assessment

FBS will be measured using the glucose oxidase method (Pars Azmun Co). HOMA-IR will be calculated using electrochemiluminescent immunoassay (ECLIA). Normal range of HOMA-IR is determined in the Iranian population by using ECLIA method with a cut-off of 2.63 [44]. TG levels will be measured using the enzymatic colorimetric method with glycerol phosphate oxidase (Pars Azmun Co). TC level will be determined using enzymatic colorimetric method with cholesterol esterase and cholesterol oxidase (Pars Azmun Co). Level of HDL-C will be determined after precipitation of apolipoprotein β with phosphotungstic acid and enzymatic colorimetric method (Pars Azmun Co). LDL-C level will be calculated using enzymatic colorimetric method (Pars Azmun Co) [45]. Serum insulin concentration will be measured using ECLIA method (Roche Diagnostics) [46]. Albumin will be measured using the enzymatic colorimetric method with cholesterol esterase and cholesterol oxidase (Pars Azmun Co). Inter- and intra-assay coefficients of variation for all metabolic measurement will be defined.

### Ultrasound Assessment

Ultrasound examination of the uterus and ovaries was performed using a 6-MHz transvaginal transducer or a 4-MHz transabdominal transducer in cases where sociocultural constraints precluded a vaginal approach for ultrasonography. Sonography was performed at the first baseline visit when the blood samples were collected. Endometrial thickness, ovarian volume, number, diameter, and distribution of the follicles were recorded. The ovaries were considered as polycystic when observed as having increased ovarian size and/or at least 10 follicular cysts measuring 2 to 9 mm [47].

### Quality of Life Assessment

The PCOSQ-50, developed by Nasiri-Amiri et al [13], was used to evaluate the quality of life of recruited participants. This questionnaire includes 50 items in the following 6 domains: psychosocial and emotional, fertility, sexual function, obesity and menstrual disorders, and coping. Items were scored based on the 5-point Likert scale.

### Sample Size

**Figure 3:**
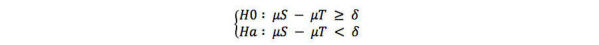
Noninferiority hypothesis.

To show that the OCs containing LNG are clinically as effective as those containing antiandrogenic progestins including DRSP, CA, EE, and DSG, we used a noninferiority hypothesis as seen in Figure 3, where μ_T_ is the mean of the test drug, μ_S_ is the mean of standard therapy, and δ is a difference of clinical importance. By rejecting the null hypothesis, we conclude that the difference between the test drug and the standard therapy is less than a clinically meaningful difference (ie, δ), and therefore the test drug is as effective as the standard therapy [48].

**Figure 4:**
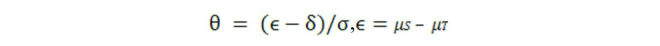
Standardized difference of observational and clinical mean difference.

**Figure 5:**
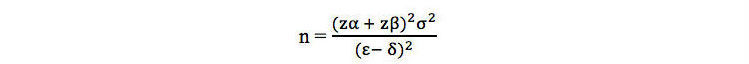
Sample size formula.

We considered 80% power, 0.05 type I error, and ϴ=0.52, where ϴ is defined as the difference of mean of test drug and standard therapy minus difference of clinical importance divided by standard division, as seen in Figure 4. Sample size was calculated from the table introduced by sample size calculations in clinical research, as seen in Figure 5 [49].

We estimated 25 samples were needed for each group, with 150 total samples needed. Considering 25% withdrawal, we will need 200 cases.

### Randomization and Blinding

A blocking or stratification random allocation with a block size of 6 using a computer-based random number generator was prepared to assign participants to treatment groups. The randomization sequence was prepared before the trial, initiated by an independent statistician. For those patients meeting the inclusion criteria and providing informed consent, the research assistant assigned the next randomization sequence according to the schedule. Although participants couldn’t be blinded to treatment type because the artifact was obvious, both clinical examiner and data analyst were blinded to participant groups during the trial.

### Statistical Analysis

Descriptive statistics will be reported appropriately; normality assumptions will be tested per case by Kolmogorov-Smirnov or Shapiro-Wilk tests. A crossover study is a longitudinal study type in which participants receive treatments in different phases. Therefore, we will use repeated measures which are correlated. We will use statistical analysis appropriate for correlated measures such as generalized estimated equations or repeated-measurement designs to find the differences between treatments. This will be done after finishing the second phase. In addition, in first phase of study, we will estimate between-group differences using generalized linear models. A washout period was taken into account although carryover effect (residual effect) will be tested at the beginning of the analysis via appropriate statistical tests like *t* tests presented by Fleiss [50]. Statistical analysis will be performed using the software package Stata version 12 (StataCorp LLC).

## Results

This trial began enrollment in February 2016, and 200 participants are currently enrolled as planned. Recruitment of participants and follow-ups are still ongoing, and preliminary results are expected to be published in 2018.

## Discussion

This study presents a protocol for a crossover randomized controlled trial to compare the effects of OCs containing LNG with products containing antiandrogenic progestins including CA, DRSP, EE, and DSG on clinical, hormonal, and metabolic findings and quality of life of reproductive-age women with PCOS. To our knowledge, this is the first trial with crossover design to compare the effects of different OCs on various clinical and biochemical aspects of PCOS. Results of this study will empower clinicians with evidence-based recommendations regarding treatment options and follow-up duration.

Different approaches have been used to treat patients with PCOS. Use of OCs is one of the most common treatment options for improving clinical and biochemical findings of PCOS [21,51]. It is well known that improvement in clinical signs of PCOS usually begins after 3 to 6 months of therapy, but because hair follicles have a half-life of up to 6 months, lifelong therapy may be needed to prevent recurrence [16,52]. In this study, patients were treated with OCs for a 6-month period in each phase.

To minimize selection bias, we enrolled patients from different settings such as professional social networks and municipal health centers, private clinics, and from among participants of the TLGS. These settings were located in different districts of Tehran. All patients who met the eligibility criteria were included in the study. Adolescent and premenopausal patients were not included in the trial because of different clinical and hormonal statuses in these periods.

An important strength of this study is its crossover design, in which the interventions under investigation are evaluated within the same patients, eliminating between-subject variability [53]. We designed this study and its methodological issues such as allocation, blinding, flow diagrams, patient preference, and carryover effects based on guidelines reported in the CONSORT statement [36]. This clinical trial is a head-to-head trial that permits patients to receive multiple treatments; hence, it can express preferences for or against particular treatments.

This study has limitations. Patients may drop out after the first intervention period and not receive a second treatment. This makes within-subject comparison impossible and is particularly important if withdrawal is related to side effects. We expect a considerable rate of loss to follow-up. To eliminate carryover effects, a washout period of 6 to 8 weeks was scheduled between treatments. Considering carryover effects of treatments across study periods can potentially distort the results obtained during the second treatment and the observed treatment effects will depend upon the order in which they will be received, hence we designed 6 treatment arms with different treatment orders. We intend to assess side effects of treatments. Considering our patients were diagnosed by AES criteria, our results may not be generalizable for those minor phenotypes diagnosed using Rotterdam criteria.

Finally, it is important to note that this project and its design are novel in the management of PCOS. If OCs containing LNG are as effective as OCs containing CA, DRSP, EE, or DSG without safety concerns, they may be recommended as a cost-effective first-line treatment for patients who suffer from the symptoms of PCOS. Therefore, we predict that the findings of this clinical trial will provide useful information for clinicians.

## Abbreviations

A4: androstenedione
AES: Androgen Excess Society
CA: cyproterone
CONSORT: Consolidated Standards of Reporting Trials
DHEAS: dehydroepiandrosterone sulfate
DRSP: drospirenone
DSG: desogestrel
ECLIA: electrochemiluminescent immunoassay
EE: ethinyl estradiol
EIA: enzyme immunoassay
ELISA: enzyme-linked immunosorbent assay
FAI: free androgen index
FBS: fasting blood sugar
FG: Ferriman-Gallwey score
FSH: follicle-stimulating hormone
HDL-C: high-density lipoprotein cholesterol
HOMA-IR: homeostatic model assessment–insulin resistance
IEMA: immunoenzymometric assay
IRMA: immunoradimetric assay
LDL-C: low-density lipoprotein cholesterol
LH: luteinizing hormone
LNG: levonorgestrel
OC: oral contraceptive
PCOS: polycystic ovary syndrome
PCOSQ-50: Health-Related Quality of Life Questionnaire for Polycystic Ovary Syndrome
RIES: Research Institute for Endocrine Studies
SHBG: sex hormone-binding globulin
TC: total cholesterol
TLGS: Tehran Lipid and Glucose Study
TG: triglycerides
tT: total testosterone
